# General hospital costs in England of medical and psychiatric care for patients who self-harm: a retrospective analysis

**DOI:** 10.1016/S2215-0366(17)30367-X

**Published:** 2017-10

**Authors:** Apostolos Tsiachristas, David McDaid, Deborah Casey, Fiona Brand, Jose Leal, A-La Park, Galit Geulayov, Keith Hawton

**Affiliations:** a Health Economics Research Centre, Nuffield Department of Population Health, University of Oxford, Oxford, UK; b Centre for Suicide Research, Department of Psychiatry, Warneford Hospital, University of Oxford, Oxford, UK; c Oxford Health NHS Foundation Trust, Oxford, UK; d Personal Social Services Research Unit, Department of Health Policy, London School of Economics and Political Science, London, UK

## Abstract

**Background:**

Self-harm is an extremely common reason for hospital presentation. However, few estimates have been made of the hospital costs of assessing and treating self-harm. Such information is essential for planning services and to help strengthen the case for investment in actions to reduce the frequency and effects of self-harm. In this study, we aimed to calculate the costs of hospital medical care associated with a self-harm episode and the costs of psychosocial assessment, together with identification of the key drivers of these costs.

**Methods:**

In a retrospective analysis, we estimated hospital resource use and care costs for all presentations for self-harm to the John Radcliffe Hospital (Oxford, UK), between April 1, 2013, and March 31, 2014. Episode-related data were provided by the Oxford Monitoring System for Self-harm and we linked these with financial hospital records to quantify costs. We assessed time and resources allocated to psychosocial assessments through discussion with clinical and managerial staff. We then used generalised linear models to investigate the associations between hospital costs and methods of self-harm.

**Findings:**

Between April 1, 2013, and March 31, 2014, 1647 self-harm presentations by 1153 patients were recorded. Of these, 1623 (99%) presentations by 1140 patients could be linked with hospital finance records. 179 (16%) patients were younger than 18 years. 1150 (70%) presentations were for self-poisoning alone, 367 (22%) for self-injury alone, and 130 (8%) for a combination of methods. Psychosocial assessments were made in 75% (1234) of all episodes. The overall mean hospital cost per episode of self-harm was £809. Costs differed significantly between different types of self-harm: self-injury alone £753 (SD 2061), self-poisoning alone £806 (SD 1568), self-poisoning and self-injury £987 (SD 1823; p<0·0001). Costs were mainly associated with the type of health-care service contact such as inpatient stay, intensive care, and psychosocial assessment. Mean costs of psychosocial assessments were £228 for adults and £392 for individuals younger than 18 years.

**Interpretation:**

If our findings are extrapolated to England, the estimated overall annual cost of general hospital management of self-harm is £162 million per year. More use of psychosocial assessment and other preventive measures, especially for young people and against self-poisoning, could potentially lower future costs at a time of major cost pressures in the NHS.

**Funding:**

National Institute for Health Research (NIHR) Collaboration for Leadership in Applied Health Research, Care Oxford at Oxford Health NHS Foundation Trust, and Department of Health.

## Introduction

Self-harm is a major public health challenge in many countries worldwide. In a 2016 report^1^ from the Multicentre Study of Self-Harm in England, age-standardised rates of hospital-presenting self-harm of 362 per 100 000 population were estimated for men and 441 per 100 000 population for women. Extrapolated to England as a whole this equates to more than 200 000 episodes every year. Despite its public health effects, limited information is published about the economic costs of self-harm. What is known about the costs of self-harm needs to be improved, both to better inform service planning and, perhaps more fundamentally, to highlight the extent of the problem and the need for services. This information could also be used to estimate potential savings in resource use and costs to the NHS through increased investment in effective measures both to prevent initial episodes of self-harm and reduce the risk of subsequent events for those who have self-harmed. Having more accurate information about the costs of self-harm is also an essential prerequisite to assessment of the cost-effectiveness of different actions, including use of psychosocial assessment and psychological therapies.

Only a small number of studies in England have attempted to cost self-harm events and most of these have focused on the costs of self-poisoning alone^2^, ^3^ rather than all types of self-harm. One exception was an analysis using data for individuals originally identified after self-harm in 1997 and followed-up to 2005, to estimate longer-term costs to health and social care services after their initial and subsequent self-harm episodes. In this study,^4^ inpatient psychiatric care accounted for two-thirds of costs in the year after the initial self-harm event. The National Institute for Health and Care Excellence (NICE) has also modelled the costs of providing initial and ongoing psychological support to adults who have self-harmed, but did not combine this with information about the initial (physical) health-care costs of treatment for self-harm.^5^

**Research in context**

**Evidence before this study**

We searched PubMed, PsycINFO, CINAHL, and EconLit for articles published in any language between Jan 1, 1988, and Jan 31, 2017, that provide evidence for the immediate costs to health-care systems of intentional self-harm, as well as the costs of psychosocial assessment. The terms “suicide” and “self-harm” were combined with “costs” and “economic evaluation” in the search process, resulting in 5354 hits and 131 articles examining cost. UK studies focused mainly on costs of self-poisoning with little focus on the costs of self-injury, similar to studies done in Belgium, Ireland, Spain, and the USA. Detailed immediate health-care costs for all methods of self-harm are rare; examples include evaluation in England (although costs were not disaggregated by method of self-harm) and Switzerland. Furthermore, limited information exists about the costs of initial psychosocial assessment in these studies. In England, NICE previously relied on NHS Reference Costs for psychiatric consultations as an indicator of the costs of psychosocial assessments after self-harm.

**Added value of this study**

To our knowledge, this is the most detailed analysis of the immediate general hospital costs of self-harm in an English hospital to date, taking account and estimating the costs of psychosocial assessment and providing different estimates of cost for different types of self-harm. Our findings show that the mean hospital cost per episode of self-harm was £809. Treatment of combined self-poisoning and self-injury is the most complex. Hospital management of children and adolescents who self-harm is more costly than that for adults. Psychosocial assessment costs a mean of £254; £392 per assessment for patients younger than 18 years and £228 for assessments for adults.

**Implications of all available evidence**

Extrapolating our findings to the whole of England would mean that the overall costs for self-harm management (assuming 75% of cases, as in the study hospital, include psychosocial assessment) in general hospitals are substantial. Using our costings, if psychosocial assessment were done for every self-harm presentation, as suggested in NICE guidelines, this would cost around £51 million per year. The study provides fundamental information that can be used to inform economic modelling analyses to better assess the potential costs and benefits of policies to address self-harm.

Robust evidence about these costs is necessary to plan service provision and assess the effect of interventions targeting self-harm. Such an intervention is the psychosocial assessment to help determine subsequent care for patients who have self-harmed.^5^, ^6^ Therefore, in this study, we aimed to calculate the costs of hospital medical care associated with a self-harm episode and the costs of psychosocial assessment, together with identification of the key drivers of these costs.

## Methods

### Study setting and self-harm data

We did a year-long retrospective longitudinal study to estimate the hospital care costs associated with self-harm in a single major general hospital, the John Radcliffe Hospital (Oxford, UK). All patients attending the hospital after self-harm between April 1, 2013, and March 31, 2014, were identified through the Oxford Monitoring System (OMS) for Self-harm. Self-harm is defined as intentional self-poisoning or self-injury, irrespective of the degree of suicidal intention or other types of motives.^7^ Data routinely collected include general self-harm method (ie, self-poisoning, self-injury, or both), specific self-harm method (eg, poisoning by specific drugs, cutting), hospital admission, patient sociodemographic characteristics (eg, age, gender, residence, and employment status), and resource use (eg, length of inpatient stay, provision of psychosocial-assessment). The OMS routinely collects data from two sources: clinicians who do psychosocial assessments and record demographic, clinical, and hospital management data on each episode, and research clerks who scrutinise emergency department electronic databases for patients who do not receive a psychosocial assessment. The sample consisted of 1647 non-fatal self-harm presentations by 1153 patients in 2013–14. These self-harm presentations were then linked with 2013–14 financial hospital records (Oxford University Hospitals NHS Trust) to obtain the costs associated with them. The pattern of self-harm in Oxford is similar to that of other centres in England where self-harm presentations are identified systematically, except that rates of self-harm in Oxford are somewhat lower than in more socioeconomically deprived areas.^1^

The Oxford Monitoring System for Self-harm has ethical approval from the NHS Health Research Authority (NRES Committee South Central, Berkshire). The OMS also has approval from the Health Research Authority Confidentiality Advisory Group under Section 251 of the NHS Act 2006 to collect non-anonymised patient details without patient consent.

### Costs of medical treatment

We obtained costs for 1623 self-harm presentations by 1140 patients (ie, 99% of the total sample). These costs included emergency department attendances, treatments received in the emergency department and hospital wards, and hospital ward and critical care unit stays. The perspective of this study is that of the hospital provider: the costs represent financial costs to the hospital rather than reimbursement values or the average costs to the NHS.

### Costs of psychosocial assessment

Psychosocial assessment by trained mental health professionals is recommended in the NICE guidelines for self-harm and includes the assessment of patients' needs and risks with the aim of determining appropriate aftercare.^5^, ^6^ For younger patients there is an emphasis on addressing safeguarding issues. NICE also recommends that all patients younger than 16 years who present to hospital after self-harm are admitted to a hospital bed.^8^ During the study period most patients who underwent psychosocial assessment were seen by a member of the emergency department psychiatric service, which is staffed by psychiatric clinical nurse specialists, psychiatrists in training, and general practitioner (GP) trainees. This service includes regular supervision sessions. During the study period, a consultant-led liaison psychiatry service was developed in the general hospital of Oxford University Hospital Trust (the OUH team), which provided assessments for a small number of adult patients, namely those admitted to non-emergency department wards.

We estimated the costs of time spent by different health-care professionals in developing, delivering, and managing psychosocial assessments for patients who self-harmed during the financial year 2013–14. The costs of the OUH liaison psychiatry service, for which it was not possible to separate psychosocial assessment costs from general hospital costs, were already included in the general hospital costs. In addition to those who delivered the assessment (ie, psychiatric clinical nurse specialists, GP trainees, and junior doctors), these assessment costs included psychiatrists' time as well as supervision and administrative support provided by administration officers. We used information from a finance manager in the Oxford Health NHS Foundation Trust, as well as extensive discussions with clinical staff, to determine the proportion of full time equivalents (FTE) devoted by each of these professionals to psychosocial assessments.

We valued time using gross mid-band salary rates (including employer's contribution to national insurance and pensions). We inflated salary costs by a factor of 20% to account for overhead costs, such as for accommodation, equipment, utility bills, and cleaning. This factor is usually used by financial administrators in NHS for overhead costs. We separately calculated the cost of psychosocial assessment for patients younger than 18 years and for those 18 years and older. We did this by proportionally allocating the FTEs of each clinician relative to the number of assessments that they delivered to each age group. We then calculated the costs per assessment by dividing total costs by the number of assessments delivered in 2013–14 for the two age groups separately. The costs of psychosocial assessments were added to all other hospital medical costs to determine total hospital costs.

### Statistical analysis

We report descriptive statistics (ie, distribution and central tendency) about the study population, methods of self-harm, related services, and costs. Non-parametric tests were used to test differences in costs between two (Mann-Whitney test) or more (Kruskal-Wallis test) independent samples. The approach we used to prepare and do the regression analysis included the specification of model structure and improvement of model fit to the data.

To specify the structure of the model (ie, which variables to include), we designed an association pathway between self-harm and costs of self-harm on the basis of available data.^9^ The association pathway (figure) included four groups of variables: total cost (ie, the sum of costs of psychosocial assessment, emergency care, inpatient care, and critical care) is the outcome variable; the general self-harm method (ie, self-poisoning alone, self-injury alone, both self-poisoning and self-injury), specific self-poisoning method, and specific self-injury method are the three variables of interest or exposure variables; hospital services received by patients, including inpatient admission, length of inpatient stay, psychosocial assessment, care from the OUH psychiatric team, and critical care were defined as intermediate variables; and morbidity, age, sex, occupational status, residence, ethnicity, and number of hospital self-harm episodes for each patient were grouped as possible confounding variables. The exposure variables can have a direct association and an indirect association with the outcome variable (figure). The indirect association is via the intermediate variables. We assumed the confounding variables were directly associated with the outcome and exposure variables. Hence, the association pathway shows that the type and method of self-harm might be associated with hospital care costs of self-harm in two ways (ie, direct association and indirect association via the intermediate variables). We specified a model that included the variable categories—outcomes, exposure, intermediate, and confounding—in a regression analysis to capture both possible association pathways between self-harm and costs. This model was specified for each of the three exposure variables, resulting in three possible model structures.

**Figure:**
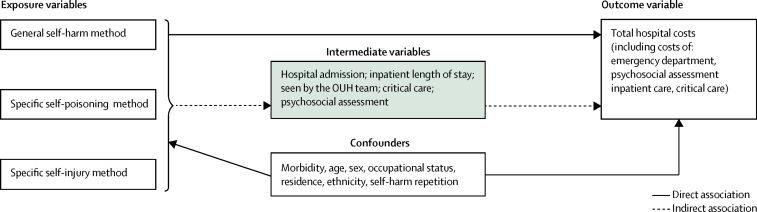
Association between self-harm and total hospital costs OUH=Oxford University Hospital.

We used generalised linear models using gamma distribution in the analysis to deal with potentially highly skewed costs resulting from many low cost episodes and a few episodes with extremely high costs. The link function of the generalised linear models was chosen based on the Akaike information criterion (AIC), the Bayesian information criterion (BIC), and the log-likelihood of the Pregibon link-test. The full regression models (ie, models with all confounders and intermediate variables as independent variables) were reduced based on Likelihood-Ratio tests to specify the structure of the model that best fitted the data. Given the low proportion of individuals with more than one repeated self-harm episode, our main analysis concerned a non-hierarchical model structure with standard errors allowing for intragroup correlation. For sensitivity analysis, we reproduced the best fitting generalised linear models using a two-level approach (ie, episodes of self-harm nested within individuals). Details on model specification are provided in the appendix. We did all analyses using STATA 13.

### Role of the funding source

The funder of the study reviewed the study proposal, awarded funding, and monitored the conduct of the study. The funders had no role in study design, data collection, data analysis, data interpretation, or writing of the report. The corresponding author had full access to all the data in the study and had final responsibility for the decision to submit for publication.

## Results

Between April 1, 2013, and March 31, 2014, 1647 self-harm presentations by 1153 patients were recorded. Of these, 1623 (99%) presentations by 1140 patients could be linked with hospital finance records. 179 (16%) of 1153 patients were younger than 18 years and 69 (6%) were older than 60 years (table 1). Most were female (719 [62%]), white (1014 [88%]), and living at home (885 [76%]). 292 (25%) were recorded as being employed, 231 (20%) reported being unemployed, 224 (19%) were students, and 164 (14%) were disabled or retired. 430 (38%) were identified as having at least one mental health condition but 346 (30%) patients were assessed as having neither a long-term physical or mental health condition (table 1).

**Table 1:** Demographic characteristics at first self-harm episode

	**n=1153**
Age (years)*	
<18	179 (16%)
18–19	84 (7%)
20–29	349 (30%)
30–39	181 (16%)
40–49	174 (15%)
50–59	117 (10%)
60–69	47 (4%)
≥70	22 (2%)
**Gender**	
Male	434 (38%)
Female	719 (62%)
**Occupational status**	
Unemployed	231 (20%)
Employed	292 (25%)
Disabled or retired	164 (14%)
Student	224 (19%)
Not known	242 (21%)
**Ethnicity**	
White	1014 (88%)
Mixed	28 (2%)
Asian	33 (3%)
Black	16 (1%)
Other	22 (2%)
Not known	40 (3%)
**Residence**	
Home not alone	708 (61%)
Home alone	177 (15%)
Lodging or hostel	99 (9%)
Institution†	40 (3%)
Not fixed or not known	129 (11%)
**Morbidity**	
No	346 (30%)
Single physical condition	104 (9%)
Single mental health condition	286 (25%)
Multiple physical conditions	31 (3%)
Multiple physical and mental conditions	144 (12%)
Not known	242 (21%)

* Age: mean 33 years (SD 16), median 29 years (range 11–97).

† Institutions include prison, long-term care, residential children care, and psychiatric hospitals.

Of 1647 episodes of self-harm, self-poisoning alone was the most frequently used method (1150 [70%]), followed by self-injury alone (367 [22%]) and combined self-poisoning and self-injury (130 [8%]) (table 2). Self-cutting (334 [67%] of 496) was the most frequently used method of self-injury, followed by hanging (46 [9%]) and jumping (14 [3%]), and a variety of other methods accounted for 97 (20%) of all self-injuries. Most self-poisoning episodes included a single drug group (748 [65%]) and paracetamol was included in 481 (42%) of all cases. 1274 (77%) self-harm episodes resulted in admission to hospital (this included admission to an emergency department short stay bed). A psychosocial assessment occurred in 1234 (75%) episodes, 1124 (88%) episodes with hospital admission and just 110 (30%) episodes without hospital admission (data not shown). For individuals admitted as inpatients, most stayed in hospital for 24–48 h (646 [51%]), followed by less than 24 h (420 [33%]), and more than 48 h (211 [17%]). Only 31 (2%) of all episodes received intensive care treatment. The vast majority of patients presented only once during the study period (942 [82%]); 129 (11%) had one additional presentation, 33 (3%) had two additional presentations, and 49 (4%) had more than two additional presentations.

**Table 2:** Type, method, and number of self-harm episodes per patient

		**n (%)**
**Type of self-harm (n=1647)**		
Self-poisoning alone		1150 (70%)
Self-injury alone		367 (22%)
Both self-poisoning and self-injury		130 (8%)
Self-injury method*(n=496)		
Cut wrist or forearm		265 (53%)
Cut elsewhere		69 (14%)
Jump from height		9 (2%)
Jump in front of moving object		5 (1%)
Hanging or asphyxiation		46 (9%)
Other method		97 (20%)
Drowning		5 (1%)
**Self-poisoning (n=1150)**		
Single drug group		
	Major tranquilisers and mood stabilisers	42 (4%)
	Benzodiazepines and other sedatives	67 (6%)
	Tricyclic antidepressants	26 (2%)
	All other antidepressants	80 (7%)
	Any paracetamol or paracetamol compounds	257 (22%)
	Other non-opiate analgesics (NSAIDs, aspirin, and compounds)	58 (5%)
	Opiate drugs only (whether prescribed or recreational)	60 (5%)
	All other substances†	142 (12%)
	Drug not known	16 (1%)
Multiple drug groups		
	Multiple categories, including any paracetamol	208 (18%)
	Multiple categories, including tricyclics	22 (2%)
	Multiple categories, including both tricyclics and any paracetamol	16 (1%)
	Multiple categories, not including tricyclics or paracetamol	156 (14%)
**Received psychosocial assessment (n=1647)**		
No		413 (25%)
Yes		1234 (75%)
**Admitted to hospital (n=1647)**		
No		370 (22%)
Emergency assessment unit		1048 (64%)
Other bed or ward		226 (14%)
Not known		3 (<1%)
**Length of hospital stay (n=1277)**		
<24 h		420 (33%)
24–48 h		646 (51%)
>48 h		211 (17%)
**Received critical care (n=1277)**		
No		1246 (98%)
Yes		31 (2%)
**Number of additional self-harm hospital episodes per patient**‡**(n=1153)**		
0		942 (82%)
1		129 (11%)
2		33 (3%)
>2		49 (4%)

* Self-injury is primary method of self-harm.

† Including prescribed medications, over the counter medications, gas, non-ingestible poisons, recreational non-opiate drugs, and alcohol.

‡ Number of repetitions per patient: mean 0·4 (SD 1·5), median 0, (range 0–22).

Psychosocial assessment was estimated to cost an average of £254 for all patients, including £392 per assessment for patients younger than 18 years and £228 for adults (appendix). Mean hospital costs were £809 (SD 1079) per self-harm episode, including psychosocial assessment costs, emergency attendance, inpatient stay, and critical care (table 3). Proportionally, more patients were admitted to a hospital bed after self-poisoning (986 [86%]) than after self-injury (179 [49%]). However, patients who self-injured stayed in hospital on average half a day longer than patients who self-poisoned, resulting in an average of £456 higher hospitalisation costs. The lowest costs in non-admitted patients were in those who had self-injured. Patients who had both self-injured and self-poisoned had the highest hospital costs (£987; SD 1823), followed by those who self-poisoned alone (£806; SD 1568), or self-injured alone (£753; SD 2061). Detailed descriptive statistics of costs are in the appendix.

**Table 3:** Hospital costs by general self-harm method and service use

	**n (%)**	**Admitted**			Total costs*		
		Admitted	Not admitted	Inpatient length of stay (days)	Admitted	Not admitted	Total costs†
All	1647 (100%)	1277 (78%)	370 (22%)	1·10 (1·78); 0–28	£963 (1899); 565 [1267]	£258 (309); £135 [356]	£809 (1709); £497 [1623]
Self-poisoning alone	1150 (70%)	986 (86%)	164 (14%)	1·03 (1·49); 0–17	£884 (1669); 563 [981]	£316 (353); £197 [156]	£806 (1568); £528 [1137]
Self-injury alone	367 (22%)	179 (49%)	188 (51%)	1·54 (3·08); 0–28	£1340 (2827); 510 [175]	£192 (211); £94 [183]	£753 (2061); £340 [358]
Both self-poisoning and self-injury	130 (8%)	112 (86%)	18 (14%)	1·00 (1·10); 0–8	£1070 (1935); 724 [111]	£446 (532); £237 [17]	£987 (1823); £667 [128]

Data are n (%), mean (SD); range, or mean (SD); median [n]. The costs of psychosocial assessment are included in the total costs; costs of 24 episodes were missing. The results of the Mann-Whitney and Kruskall-Wallis tests were confirmed by generalised linear models with log family and identity link function.

* Total costs were significantly (ie, self-poisoning alone p<0·0001, self-injury alone p<0·0001, and both self-poisoning and self-injury p<0·0003) different between admitted and not-admitted episodes in all self-harm methods based on the Mann-Whitney statistical test.

† Total costs are significantly (ie, p<0·0001) different between self-harm methods based on the Kruskall-Wallis statistical test.

The generalised linear models with γ distribution and identity link function had the best fit to the data for each of the three regression models (ie, self-harm type, self-injury method, or self-poisoning method as the exposure variable; appendix p 1; table 4). Model 1 shows that self-harm patients who received a psychosocial assessment had on average £270 (95% CI 223 to 316) higher costs (including the costs of psychosocial assessment) than non-assessed patients. Those who were admitted to hospital had on average £155 (97 to 214) higher costs than non-admitted patients, £161 (114 to 207) higher for each inpatient day and £6180 (3724 to 8635) higher if they received treatment in intensive care. Age, which was the only confounder contributing to the fit of the model, had a U-shape association with costs. Costs were initially reduced from age 13 years by £101 (−179 to −22) per 10 additional life-years; from the age of 39 years costs increased by £13 (2 to 23) per 10 additional life years after adjusting for the other covariates (data not shown). After adjusting for hospital use (intermediate) variables and age, self-poisoning alone was £120 (22 to 218) more costly than self-injury alone and the combination of self-poisoning and self-injury was £74 (26 to 121) more costly. Model 2 shows that among patients who self-injured, no cost differences were found across self-injury methods after adjusting for psychosocial assessment, hospital admission, inpatient length of stay, and treatment by the OUH team. Model 3 shows that episodes of patients who self-poisoned using multiple medicines, including tricyclic antidepressants and paracetamol, were a mean of £56 (−95 to −17) less costly than episodes involving major tranquillisers and mood stabilisers after adjusting for psychosocial assessment, hospital admission, inpatient length of stay, and treatment in intensive care. The results of the full regression models are in the appendix (pp 2–4). The results of the sensitivity analysis (ie, when specifying multilevel models) are also in the appendix (p 6). The coefficients and p values from the multi-level models were very similar to those presented in table 4.

**Table 4:** Adjusted association of self-harm with total hospital costs

		**Model 1 Exposure: self-harm method *b* (SE); p value; 95% CI**	**Model 2 Exposure: self-injury method *b* (SE); p value; 95% CI**	**Model 3 Exposure: self-poisoning method *b* (SE); p value; 95% CI**
Constant		£280 (70); <0·0001; 142 to 418	£102 (17); <0·0001; 69 to 136	£227 (60); <0·0001; 109 to 345
Method of self-harm (ref cat self-injury alone)				
	Self-poisoning alone	£74 (24); 0·0022; 26 to 121	··	··
	Both self-poisoning and self-injury	£120 (50); 0·017; 22 to 218	··	··
Self-injury method (ref cat cut wrist)				
	Cut elsewhere	··	–£19 (19); 0·31; −56 to 18	··
	Jump from height	··	£41 (55); 0·45; −66 to 149	··
	Jump in front of moving objects	··	–£25 (18); 0·16; −60 to 10	··
	Hanging or asphyxiation	··	£59 (33); 0·072; −5 to 123	··
	Other method	··	£29 (21); 0·17; −13 to 70	··
	Drowning	··	£85 (192); 0·65; −292 to 462	··
Self-poisoning method (ref cat tranquillisers and mood stabilisers)				
	Benzodiazepines and other sedatives	··	··	£42 (93); 0·64; −139 to 224
	Tricyclic antidepressants	··	··	£87 (90); 0·33; −89 to 264
	All other antidepressants	··	··	£32 (34); 0·34; −35 to 99
	Any pure paracetamol (compounds)	··	··	£54 (32); 0·08; −8 to 116
	Other non-opiate analgesics	··	··	£13 (47); 0·78; −80 to 106
	Opiate drugs only	··	··	£7 (25); 0·77; −42 to 57
	All other substances	··	··	£40 (44); 0·36; −46 to 126
	Drug not known	··	··	£73 (102); 0·47; −127 to 273
	Multiple including any paracetamol	··	··	£75 (45); 0·099; −14 to 163
	Multiple including tricyclics	··	··	£57 (47); 0·22; −35 to 148
	Multiple including tricyclics and paracetamol	··	··	–£56 (20); 0·0051; −95 to −17
	Multiple excluding tricyclics and paracetamol	··	··	£15 (41); 0·71; −64 to 95
Age (10 years)		–£101 (40);0·012; −179 to −22	··	
(Age [10 years])^2^		£13 (6); 0·022; 2 to 23	··	
Employed (ref cat unemployed)		··	··	£1 (44); 0·98; −85 to 86
Disabled or retired (ref cat unemployed)		··	··	£60 (43); 0·16; −25 to 145
Student (ref cat unemployed)		··	··	£197 (57); 0·0006; 85 to 308
Not known (ref cat unemployed)		··	··	–£7 (43); 0·87; −90 to 77
Home alone (ref cat home not alone)		··	··	–£51 (43); 0·23; −136 to 33
Living in lodging or hostel (ref cat home not alone)		··	··	–£26 (64); 0·69; −152 to 100
Living in institution (ref cat home not alone)		··	··	–£125 (46); 0·0062; −214 to −35
Not fixed or not known (ref cat home not alone)		··	··	–£136 (34); <0·0001; −202 to −69
Assessed (ref cat not assessed)		£270 (24); <0·0001; 223 to 316	£296 (34); <0·0001; 230 to 362	£240 (49); <0·0001; 143 to 336
Admitted (ref cat not admitted)		£155 (30); <0·0001; 97 to 214	£219 (45); <0·0001; 130 to 307	£97 (31); 0·0016; 37 to 157
Length of stay (days)		£161 (24); <0·0001; 114 to 207	£255 (45); <0·0001; 166 to 343	£86 (24); 0·0003; 39 to 132
Seen by the OUH team (ref cat no OUH team)		£1809 (1265); 0·15; −670 to 4289	£7786 (2345); 0·0009; 3189–12 382	··
Received critical care (ref cat no critical care)		£6180 (1253); <0·0001; 3724 to 8635	··	£7133 (1216); <0·0001; 4749 to 9516
n episodes (n patients)		1623 (1140)	485 (347)	1137 (857)
AIC/BIC		23 841/23 895	6882/6928	16 919/17 045

Data are *b* (SE), which represents mean cost; p value; 95% CI, unless otherwise specified. These are the reduced models after including only the intermediate variables and confounders that improve the model fit to the data; generalised linear model with γ distribution and identity link with standard errors allowing for intragroup correlation. ref cat=reference category. AIC=Akaike information criterion. BIC=Bayesian information criterion. OUH=Oxford University Hospital Trust team.

## Discussion

Our findings show that the mean hospital cost per episode of self-harm was £809. These costs are mainly driven by the health-care services used after self-harm, such as admission to hospital, inpatient length of stay, treatment in intensive care, and psychosocial assessment. Holding all else constant, costs differed significantly between different types of self-harm, with self-injury alone being associated with the lower costs, followed by self-poisoning alone (£74 higher than self-injury), and self-injury and self-poisoning combined (£120 higher than self-injury alone).

Although for patients admitted to a hospital bed, self-poisoning was associated with a shorter inpatient stay than self-injury, we have shown that the treatment of patients who self-poisoned was more complex and more costly per hospital day than the treatment of patients who self-injured. However, we found no significant differences in costs within the specific types of self-injury methods and self-poisoning methods.

We also estimated the mean cost of psychosocial assessment to be £254 but found a difference of £164 between psychosocial assessment for patients younger than 18 years (£392) and the mean cost for adults (£228). This reflects the fact that children and adolescents often have complex needs that require more time for assessment compared with adult patients. This is likely to be determined by greater need to interview other informants, especially parents, and to address safeguarding issues. Furthermore, the incidence of self-harm in people younger than 18 years is particularly high.^10^

This study is to our knowledge the most detailed analysis of the immediate general hospital costs of self-harm in England to date, taking account of the use of psychosocial assessment and providing different estimates of cost for different types of self-harm. Previous analyses^2^, ^3^ from the turn of the millennium have focused on the costs of self-poisoning alone, including the costs of antidepressant overdoses,^11^ in which hospital costs of tricyclic overdoses were significantly greater than those of SSRI overdoses.^12^ Analysis of self-poisoning episodes in a small number of English hospitals^13^, ^14^ previously highlighted variability in care management with wide differentials in cost per episode, for instance ranging from £312 to £577 (not including psychosocial assessment; 2016 prices).^14^ Another analysis, rather than estimating actual costs, made use of specific NHS Reference cost tariffs for emergency medicine to different clinical pathways after paracetamol poisoning to estimate UK-wide annual hospital costs to be in the region of £51 million.^15^

Outside of the UK, few detailed studies on the costs of all-causes of self-harm are available. In Switzerland, the 1-year costs of all self-harm events at two hospitals in Basel in 2003^16^ were estimated. Older age and more lethal methods (eg, hanging, drowning or jumping) were associated with significantly higher costs, with median costs per case of £3437. A 2012^17^ analysis of self-poisonings in a Belgian hospital estimated the mean cost of self-poisoning to be similar in magnitude to that seen in our study, at £810. Mean hospital costs per poisoning (both unintentional and intentional) estimated in one hospital in Madrid, Spain, also indicated that costs rise with age, but no precise median or mean costs per case were provided.^18^ Mean costs of just 17 self-harm cases (seven of which were self-injury and ten poisonings) were £4142 in one hospital in São Paulo, Brazil.^19^ In the USA, large patient and injury databases have also been used to look at the costs of self-harm. National level costs for non-fatal self-harm events were reported in 2016 to be a mean of £6061, but this includes productivity losses in addition to medical costs.^20^ Another US analysis from 2016 has estimated the hospitalisation costs per suicide-related poisoning and alcohol overdose to be $4129.^21^ The hospital costs estimates reported in international literature as well as in this study should be considered cautiously from an international perspective as the organisation of hospital services delivered for self-harm will vary between countries.

To our knowledge, our calculation of the psychosocial assessment costs is the first detailed estimate of assessment costs in England. NICE guidelines on self-harm management included a cost calculation of psychosocial assessment based on reference costs (2009–10) of first time face-to-face consultations with mental health consultants in a community setting.^8^ Our estimates of psychosocial assessment costs were £36 lower for patients younger than 18 years (£392 *vs* £356) and £2 lower for adults (£228 *vs* £226) than the costs provided by NICE after inflating to 2013–14 values using the Inflation Hospital Community Health Services Index.^8^ This means that the costs of the Oxford model of psychosocial assessment were very close to the mean psychosocial assessment costs in England in 2013–14 as estimated by NICE, which supports the generalisability of the results of the present study.

Although the rate of self-harm in Oxford is somewhat lower than in some other centres,^1^ the pattern of self-harm in this study is similar in terms of the characteristics of patients and methods of self-harm to that found in other hospitals in England.^22^ A large observational study^23^ of self-harm in 32 English hospitals in 2010–11 showed that the proportion of self-harm patients who were admitted to general hospitals varied between 22% and 85% of those presenting and the proportion of episodes in which a psychosocial assessment was done varied between 22% and 88%. Considering these large variations and the recent increased tendency to admit self-harm patients to hospital and to do psychosocial assessment (as suggested in NICE guidelines), we believe that our study provides a first reasonable contemporary estimation of the mean cost per self-harm episode in England. If we extrapolate our findings to England, the estimated costs of self-harm in general hospitals are £161·8 million per year (ie, 200 000 presentations multiplied by £809).

Psychosocial assessment, followed by appropriate treatment and care, potentially provides a way of reducing the risk of future self-harm^24^, ^25^ and is therefore recommended in NICE clinical guidelines. Using our costings, if psychosocial assessment was done for every self-harm presentation, this would cost around £51 million per year. Further evidence from economic evaluation studies is needed to show the potential savings that might result from greater use of psychosocial assessment after self-harm. Such studies are challenging and appropriate study designs should be adopted.^26^

Our estimate of the costs of hospital management of self-harm provides fundamental information for future economic modelling analyses to evaluate the potential costs and benefits of policies for self-harm. These include psychosocial assessment, aftercare interventions, and prevention initiatives. Evidence from such analyses could support policy makers in trying to achieve reduction in suicide.^27^

The strengths of this study include use of a large dataset of good data quality, the advanced regression modelling approach, and the precision of the reported costs of psychosocial assessment. The limitations of this study include the lack of a breakdown by mental health conditions, the single hospital setting and the limitation of its scope to the hospital costs after self-harm. Future research might explore the medical costs throughout the care pathways by including ambulance costs, costs borne by the patient and their families, and after-care costs in several settings across England. It will also be important to use follow-up data for patients with self-harm to explore their subsequent use of health and social care services, as well as broader outcomes such as the effect on their quality of life.

Hospital management of self-harm involves substantial costs. According to our findings the mean cost is £809 per self-harm episode, including the costs of psychosocial assessment. Given the size of the problem of self-harm nationally in England this translates into approximately £162 million per year from the NHS budget to treat people with self-harm presentations to general hospitals. This highlights the need for effective methods of preventing of self-harm and reducing the extent to which it is repeated.
