# Supplementary material for: General hospital costs in England of medical and psychiatric care for patients who self-harm: a retrospective analysis
Source: Lancet Psychiatry. 2017 Oct;4(10):759–67. doi: 10.1016/S2215-0366(17)30367-X (PMC5614771; doi:10.1016/S2215-0366(17)30367-X)
Supplement: Supplementary appendix [file mmc1.pdf]

THE LANCET
Psychiatry

Supplementary appendix

This appendix formed part of the original submission and has been peer reviewed. We post it as supplied by the authors.

Supplement to: Tsiachristas A, McDaid D, Casey D, et al. General hospital costs in England of medical and psychiatric care for patients who self-harm: a retrospective analysis. *Lancet Psychiatry* 2017; published online Sept 7. http://dx.doi.org/10.1016/S2215-0366(17)30367-X.

Appendix 1 Regression modelling strategy and its results

First, we used generalised linear models (GLMs) with gamma distribution and standard errors adjusted for clustering of self-harm episodes in patients and assessed whether an identity or a log link function was better fitting the data based on the Akaike information criterion (AIC), the Bayesian information criterion (BIC), and the log-likelihood of the link-test. The link function that resulted in a lower value for these criteria was selected to be used in the regression models (see Supplementary Table 1.1).

Supplementary Table 1.1 Goodness-of-fit results of different link functions

	general self-harm method		Self-injury method		Self-poisoning method	
Link	ID	Log	ID	Log	ID	Log
AIC	23856·28	23893·022	6899·91	6947·038	16941·13	16941·61
BIC	24018·043	24054·78	7025·43	7072·56	17142·58	17143·062
log-likelihood of link-test	-13744·15	-13674·59	-4179·54	-4177·92	-9471·85	-9346·25

Second, the full regression models (i.e. models with all confounders and intermediate variables aside the exposure variable) were reduced based on Likelihood-Ratio tests to specify the structure of the model that best fitted the data. The results of the full regression models are presented in Supplementary Tables 1.2 to 1.4. The choice of using non-hierarchical GLMs in the main analysis was based on the following: a) the dataset was largely unbalanced with only 7% (n=82) of all patients (n=1153) having two or more repetitions in the study period, b) the number of repetitions was not a predictor of health resource utilisation (i.e. hospital admission (p=0.457), inpatient length of stay (p=0.473), provision of psychosocial assessment (p=0.857)), c) the intra-cluster correlation coefficient (ICC) was 0·174 (95%CI: 0·169;0·179) indicating that there was weak correlation between the per episode costs of a patient.

Third, as sensitivity analysis, we report the best fitting GLM models using a hierarchical structure, i.e. allowing correlation among total hospital costs of self-harm episodes from the same individuals (Supplementary Table 1.5).

Analyses were carried out using STATA 13.

Supplementary Table 1.2 Association of self-harm method with total hospital costs of episode (full models)

**Variable**	**Full model (direct relation) b (se) |p-value| [95% CI]**	**Full model (direct & indirect relation) b (se) |p-value| [95% CI]**
Constant	1205 (258) |<0·0001| [698;1711]	251 (93) |0·0067| [70;433]
Method of self-harm (ref. cat.: Self-injury)		
Self-poisoning	83 (45) |0·067| [-6;172]	56 (24) |0·017| [10;102]
Both	98 (97) |0·31| [-92;287]	85 (47) |0·072| [-8;178]
Morbidity (ref. cat.: No Morbidity)		
Single physical condition	-86 (51) |0·094| [-186;15]	-29 (47) |0·53| [-120;62]
Single psychiatric condition	85 (70) |0·22| [-51;222]	46 (27) |0·09003| [-7;98]
Multiple physical conditions	-166 (113) |0·14| [-387;55]	120 (139) |0·39| [-153;393]
Multiple physical and psychiatric conditions	208 (174) |0·23| [-133;550]	-9 (38) |0·81| [-83;65]
Not known	-245 (69) |0·00038| [-380;-111]	13 (14) |0·33| [-14;40]
Female (ref. cat.: Male)	-102 (53) |0·054| [-206;2]	-24 (21) |0·25| [-66;18]
Age (10 years)	-382 (159) |0·017| [-694;-69]	-67 (38) |0·078| [-141;8]
Age (10 years) squared	71 (24) |0·0034| [23;118]	9 (5) |0·097| [-2;19]
Occupational status (ref. cat.: Unemployed)		
Employed	-80 (68) |0·23| [-213;52]	-24 (39) |0·54| [-101;53]
Disabled/retired	40 (86) |0·64| [-129;209]	32 (57) |0·58| [-81;144]
Student	176 (96) |0·067| [-12;364]	94 (46) |0·041| [4;183]
Not known	-97 (76) |0·206| [-246;53]	22 (44) |0·62| [-65;108]
Residence (ref. cat.: home not alone)		
Home alone	-120 (62) |0·053| [-241;2]	-28 (51) |0·58| [-128;72]
Lodging/hostel	-40 (84) |0·63| [-204;124]	-51 (45) |0·26| [-139;38]
Institution	-67 (95) |0·47| [-253;119]	-40 (37) |0·27| [-112;32]
Not fixed/ not known	-144 (71) |0·043| [-283;-5]	-61 (33) |0·064| [-125;4]
Ethnicity (ref. cat.: White)		
Mixed	-127 (63) |0·044| [-250;-3]	-27 (21) |0·204| [-69;15]
Asian	-163 (68) |0·017| [-298;-29]	-28 (28) |0·32| [-83;27]
Black	64 (127) |0·61| [-184;312]	-26 (85) |0·76| [-193;142]
Other	-176 (67) |0·0086| [-306;-45]	83 (105) |0·42| [-122;288]
Not known	726 (546) |0·18| [-344;1795]	-59 (14) |<0·0001| [-86;-31]
Number of self-harm repetitions		-1 (1) |0·71| [-3;2]
Admitted (ref. cat.: Not admitted)		154 (28) |<0·0001| [100;209]
Assessed (ref. cat.: Not assessed)		255 (40) |<0·0001| [177;333]
Length of stay (days)		154 (22) |<0·0001| [110;198]
Seen by the OUH team (ref. cat.: No OUH team)		1833 (1269) |0·14| [-653;4320]
Received critical care (ref. cat.: No critical care)		6201 (1253) |<0·0001| [3745;8658]
n episodes (n patients)	1623 (1140)	1623 (1140)
AIC/BIC	24569/24704	23856/24018

Ref. cat.: reference category; Akaike information criterion (AIC), Bayesian information criterion (BIC); the analysis was performed with Generalised Liner Models using gamma distribution and identity link

Supplementary Table 1.3 Association of self-injury method with total hospital costs of episode (full models)

Variable	Full model (direct relation) b (se) |p-value| [95% CI]	Full model (direct & indirect relation)
Constant	1505 (371) |<0·0001| [778;2232]	214 (72) |0·0029| [73;356]
Self-injury method (ref. cat.: cut wrist)		
Cut elsewhere	-37 (21) |0·072| [-78;3]	-14 (13) |0·27| [-40;12]
Jump from height	3944 (2915) |0·17| [-1770;9657]	43 (55) |0·43| [-64;150]
Jump in front of moving objects	-92 (1182) |0·93| [-2409;2226]	-9 (13) |0·52| [-35;18]
Hanging/asphyxiation	83 (58) |0·15| [-31;197]	73 (30) |0·015| [14;132]
Other method	210 (105) |0·046| [4;415]	41 (17) |0·015| [8;75]
Drowning	3 (368) |0·99| [-719;725]	110 (224) |0·62| [-328;548]
Morbidity (ref. cat..: No Morbidity)		
Single physical condition	-62 (140) |0·65| [-335;212]	148 (172) |0·39| [-189;484]
Single mental condition	13 (105) |0·89| [-192;219]	87 (39) |0·026| [11;164]
Multiple physical conditions	-47 (383) |0·902| [-798;704]	317 (399) |0·42| [-466;1099]
Multiple physical and mental conditions	-108 (103) |0·29| [-309;94]	16 (60) |0·78| [-101;134]
Not known	-380 (97) |<0·0001| [-571;-190]	16 (20) |0·41| [-23;56]
Female (ref. cat..: Male)	-117 (66) |0·079| [-247;14]	-14 (29) |0·63| [-71;43]
Age (10 years)	-594 (279) |0·033| [-1140;-47]	-73 (40) |0·068| [-152;5]
Age (10 years) squared	111 (45) |0·015| [21;200]	10 (6) |0·101| [-2;22]
Residence (ref. cat.: home not alone)		
Home alone	-164 (117) |0·15| [-393;64]	-34 (26) |0·19| [-84;17]
Lodging/hostel	-22 (124) |0·85| [-266;221]	-17 (24) |0·48| [-65;31]
Institution	-121 (96) |0·21| [-310;68]	-14 (17) |0·43| [-47;20]
Not fixed/ not known	-124 (105) |0·24| [-330;83]	0 (20) |0·99| [-40;40]
Ethnicity (ref. cat.: White)		
Mixed	-20 (35) |0·56| [-89;49]	20 (38) |0·58| [-53;94]
Asian	32 (424) |0·94| [-800;863]	-4 (22) |0·87| [-47;40]
Black	58 (156) |0·71| [-248;364]	-190 (77) |0·014| [-342;-38]
Other	40 (198) |0·84| [-348;428]	300 (163) |0·066| [-20;620]
Not known	-18 (147) |0·903| [-306;270]	-47 (14) |0·00071| [-74;-20]
Number of self-harm repetitions	1 (2) |0·804| [-4;5]	-1 (2) |0·58| [-4;2]
Admitted (ref. cat.: Not admitted)		209 (45) |<0·0001| [121;298]
Assessed (ref. cat.: Not assessed)		264 (39) |<0·0001| [187;341]
Length of stay (days)		255 (44) |<0·0001| [169;341]
Seen by the OUH team (ref. cat.: No OUH		5968 (3728) |0·109| [-1339;13275]
Received critical care (ref. cat.: Not received		3848 (4347) |0·37| [-4671;12367]
n episodes (n patients)	485 (347)	485 (347)
AIC/BIC	7150/7254	6900/7025

Ref. cat.: reference category; Note: Occupational status was excluded by the models because of collinearity; Akaike information criterion (AIC), Bayesian information criterion (BIC); the analysis was performed with Generalised Liner Models using gamma distribution and identity link

Supplementary Table 1.4 Association of self-poisoning method with total hospital costs of episode (full models)

**Variable**	**Full model (direct relation) b (se) |p-value| [95% CI]**	**Full model (direct & indirect relation) b (se) |p-value| [95% CI]**
Constant	1180 (321) |0·00023| [550;1809]]	391 (123) |0·0014| [151;631]
Self-poisoning method (ref. cat.: Major tranquilisers. & mood stabilisers)		
Benzodiazepines & other sedatives	7 (52) |0·89| [-95;109]	30 (60) |0·61| [-87;147]
Tricyclic antidepressants	831 (493) |0·092| [-134;1797]	67 (93) |0·46| [-115;250]
All other antidepressants	292 (184) |0·11| [-69;654]	17 (38) |0·66| [-59;92]
Any pure paracetamol (compounds)	134 (49) |0·0058| [39;229]	51 (35) |0·14| [-18;120]
Other non-opiate analgesics	141 (149) |0·34| [-150;433]	-6 (63) |0·92| [-128;117]
Opiate drugs only	53 (63) |0·39| [-70;176]	5 (34) |0·87| [-61;71]
All other drugs	140 (69) |0·042| [5;275]	37 (44) |0·405| [-50;124]
Drug not known	1006 (605) |0·096| [-179;2191]	63 (85) |0·45| [-104;231]
Multiple including any paracetamol	144 (65) |0·027| [16;272]	74 (46) |0·109| [-16;164]
Multiple including tricyclics	1457 (743) |0·049| [1;2913]	23 (52) |0·66| [-80;125]
Multiple including tricyclics & paracetamol	235 (322) |0·46| [-395;866]	-86 (36) |0·018| [-157;-15]
Multiple excluding tricyclics & paracetamol	137 (90) |0·12| [-40;314]	5 (37) |0·901| [-68;77]
Morbidity (ref. cat..: No Morbidity)		
Single physical condition	-52 (49) |0·29| [-148;45]	-44 (42) |0·29| [-126;38]
Single mental condition	83 (58) |0·15| [-31;197]	41 (34) |0·22| [-25;107]
Multiple physical conditions	-104 (92) |0·26| [-284;77]	88 (145) |0·54| [-196;372]
Multiple physical and mental conditions	297 (176) |0·091| [-47;642]	9 (44) |0·84| [-77;95]
Not known	-234 (83) |0·005| [-397;-71]	19 (41) |0·64| [-62;100]
Female	-75 (49) |0·12| [-170;21]	-41 (19) |0·034| [-79;-3]
Age (10 years)	-397 (196) |0·043| [-782;-13]	-100 (53) |0·059| [-203;4]
Age (10 years) squared	59 (29) |0·044| [2;117]	14 (7) |0·045| [0;27]
Residence (ref. cat.: Home not alone)		
Home alone	-9 (54) |0·86| [-114;96]	0 (47) |0·99| [-92;91]
Lodging/hostel	165 (98) |0·092| [-27;357]	26 (44) |0·55| [-61;112]
Institution	187 (102) |0·068| [-14;388]	152 (64) |0·018| [26;279]
Not fixed/not known	-1 (74) |0·98| [-146;144]	19 (58) |0·74| [-94;132]
Ethnicity (ref. cat.: White)		
Mixed	-147 (65) |0·023| [-274;-21]	-56 (44) |0·205| [-142;30]
Asian	-88 (65) |0·17| [-216;40]	-20 (70) |0·77| [-157;116]
Black	73 (137) |0·59| [-196;342]	-136 (56) |0·015| [-246;-26]
Other	-119 (78) |0·12| [-271;33]	-147 (38) |0·0034| [-222;-72]
Not known	35 (117) |0·76| [-195;265]	-3 (50) |0·94| [-101;94]
Number of self-harm repetitions	0 (4) |0·91| [-9;8]	2 (3) |0·45| [-3;7]
Admitted (ref. cat.: Not admitted)		95 (30) |0·0013| [37;153]
Assessed (ref cate: Not assessed)		264 (57) |<0·0001| [152;376]
Length of stay (days)		86 (25) |0·00049| [38;135]
Seen by the OUH team (ref. cat.: No OUH team)		-207 (136) |0·12| [-474;60]
Received critical care (ref. cat.: Not received critical care)		7241 (1210) |<0·0001| [4870;9612]
n episodes (n patients)	1137 (857)	1137 (857)
AIC/BIC	17328/17504	16941/17143

Ref. cat.: reference category; *the saturated model included the variables: type of self-harm, morbidity, gender, age, age squared, occupational status, residence, ethnicity, number of repetitions; Akaike information criterion (AIC), Bayesian information criterion (BIC); the analysis was performed with Generalised Liner Models using gamma distribution and identity link

Supplementary Table 1.5 Results of the sensitivity analysis when specifying multi-level regression models

**Intermediate variables and confounders**	**Model 1 Exposure: self-harm method b (se) |p-value| [95% CI]**	**Model 2 Exposure: self-injury method b (se) |p-value| [95% CI]**	**Model 3 Exposure: self-poisoning method b (se) |p-value| [95% CI]**
Constant	270 (55) |<0·0001| [162;379]	104 (17) |<0·0001| [70;137]	227 (60) |0·00019| [107;346]
Method of self-harm (ref. cat.: Self-injury alone)			
Self-poisoning alone	79 (29) |0·00709| [22;137]		
Both self-poisoning and self-injury	124 (36) |0·0013| [48;200]		
Self-injury method (ref. cat.: cut wrist)			
Cut elsewhere		-20 (19) |0·28| [-57;17]	
Jump from height		41 (56) |0·46| [-68;151]	
Jump in front of moving objects		-25 (18) |0·15| [-60;10]	
Hanging/asphyxiation		58 (38) |0·12| [-17;134]	
Other method		27 (20) |0·17| [-12;65]	
Drowning		85 (180) |0·63| [-269;439]	
Self-poisoning method (ref. cat.: Tranquilisers & mood stabilisers)			
Benzodiazepines & other sedatives			43 (71) |0·55| [-97;182]
Tricyclic antidepressants			88 (68) |0·19| [-46;221]
All other antidepressants			32 (48) |0·506| [-62;126]
Any pure paracetamol (compounds)			54 (33) |0·098| [-10;118]
Other non-opiate analgesics			13 (43) |0·759| [-71;97]
Opiate drugs only			7 (30) |0·809| [-51;66]
All other drugs			40 (36) |0·277| [-32;111]
Drug not known			74 (99) |0·457| [-121;268]
Multiple including any paracetamol			75 (54) |0·164| [-30;180]
Multiple including tricyclics			57 (73) |0·435| [-86;199]
Multiple including tricyclics & paracetamol			-56 (22) |0·012| [-100;-12]
Multiple excluding tricyclics & paracetamol			15 (38) |0·689| [-60;90]
Age (10 years)	-94 (31) |0·0021| [-155;-34]		
Age (10 years) squared	12 (4) |0·0044| [4;20]		
Employed (ref. cat.: Unemployed)			1 (39) |0·97| [-76;78]
Disabled/retired (ref. cat.: Unemployed)			61 (46) |0·18| [-29;150]
Student (ref. cat.: Unemployed)			197 (48) |<0·0001| [103;291]
Not Known (ref. cat.: Unemployed)			-6 (47) |0·89| [-98;85]
Home alone			-52 (45) |0·25| [-139;36]
Living in Lodging/hostel			-26 (77) |0·73| [-176;125]
Living in institution			-125 (45) |0·00601| [-214;-36]
Not fixed/not known			-135 (37) |0·00023| [-208;-63]
Assessed (ref. cat.: Not assessed)	276 (27) |<0·0001| [223;329]	300 (46) |<0·0001| [209;391]	240 (49) |<0·0001| [143;336]
Admitted (ref. cat.: Not admitted)	157 (31) |<0·0001| [96;217]	214 (63) |0·00069| [91;339]	97 (31) |0·00405| [37;157]
Length of stay (days)	157 (31) |<0·0001| [96;217]	248 (55) |<0·0001| [140;357]	86 (24) |0·00015| [39;132]
Seen by the OUH team (ref. cat.: No OUH team)	1878 (1639) |0·25| [-1334;5089]	7790 (2526) |0·00204| [2838;12741]	
Received critical care (ref. cat.: No critical care)	6133 (1303) |<0·0001| [3579;8687]		7133 (1216) |<0·0001| [4749;9516]
n episodes (n patients)	1623 (1140)	485 (347)	1137 (857)

Note: the analysis was performed with Multilevel Generalised Liner Models using gamma distribution and identity link

Appendix 2. Cost calculation of psychosocial assessment

**Assumptions**	**Value**					
Overhead	20%					
Full-time hours/week	37·5					
Full-time weeks/year	44					
Whole Time Equivalents in hours per year	1650					
Hours/year for wash-up	65					
Hours/year for monthly	12					
Admin minutes/assessment	30					
Consultant time for supervision (JD&GP) in minutes	15					
Junior doc hours/assessment (hours)	3					
GP trainee hours/assessment (hours)	3					
Nurse Band 7 hours/assessment (adult)	3					
Nurse Band 7 hours/assessment (under 18)	5					
Consultant time for supervision (nurse7 under 18) in minutes	15					
**Personnel costs**	WTE	proportion WTE for self-harm	WTE for self-harm	Mid-Band salary	Overhead	Total costs
Nurse Band 7	4	0·75	3·00	£50070	£10014	£180250
Nurse Band 8	1	0·19	0·19	£58607	£11721	£13187
Admin & Clerical Band 4	1	0·37	0·37	£26949	£5390	£12093
GP Trainee	1	0·5	0·50	£74000	£14800	£44400
Junior Doctor	1	0·5	0·50	£60000	£12000	£36000
Consultant Psychiatrist	1	0·17	0·17	£141133	£28227	£28073
Total personnel costs						*£314002*
**Personnel costs (adults)**			WTE for adults	Mid-Band salary	Overhead	Total costs
Nurse Band 7			2·08	£50070	£10014	£125003
Nurse Band 8			0·13	£58607	£11721	£9145
Admin & Clerical Band 4			0·31	£26949	£5390	£10152
GP Trainee			0·50	£74000	£14800	£44400
Junior Doctor			0·50	£60000	£12000	£36000
Consultant Psychiatrist			0·07	£141133	£28227	£11753
Total personnel costs						*£236453*
**Personnel costs (under 18)**			WTE for adults	Mid-Band salary	Overhead	Total costs
Nurse Band 7			0·92	£50070	£10014	£55247
Nurse Band 8			0·06	£58607	£11721	£4042
Admin & Clerical Band 4			0·06	£26949	£5390	£1940
GP Trainee			0·00	£74000	£14800	£0
Junior Doctor			0·00	£60000	£12000	£0
Consultant Psychiatrist			0·10	£141133	£28227	£16320
Total personnel costs						*£77549*
Total cost/assessment (all)						**£254**
Total cost/assessment (adults)						***£228***
Total cost/assessment (under 18)						***£392***

Appendix 3. Results of the descriptive analysis of costs

Variable	Total costs Mean (SD) {median} [n]	Variable	Total costs Mean (SD) {median} [n]
Age***	***	Length of hospital stay***	
<18	999 (932) {726} [229]	<24 hours	496 (1299) {388} [763]
18-19	530 (360) {506} [95]	24-48 hours	738 (611) {621} [650]
20-29	560 (1422) {404} [543]	>48 hours	2165 (3622) {1020} [210]
30-39	611 (781) {497} [243]	Seen by the OUH team***	
40-49	1005 (2296) {473} [274]	No	676 (1027) {491} [1581]
50-59	976 (1913) {562} [155]	Yes	5790 (6982) {3677} [42]
60-69	2090 (4365) {695} [55]	Assessed***	
70 and older	1350 (1661) {820} [29]	No	313 (1197) {128} [390]
Gender*		Yes	966 (1814) {574} [1233]
Male	942 (2114) {513} [562]	Received intensive care***	
Female	738 (1445) {485} [1061]	No	650 (965) {488} [1592]
Occupational status***		Yes	8945 (6222) {6859} [31]
Unemployed	821 (1807) {525} [321]	Self-injury method*	
Employed	808 (1835) {522} [378]	Cut wrist/forearm	568 (826) {383} [262]
Disabled/retired	1073 (2007) {621} [289]	Cut elsewhere	709 (1774) {344} [69]
Student	937 (878) {633} [281]	Jump from height	5132 (8776) {545} [9]
Not known	481 (1668) {151} [354]	Jump in front of moving object	2084 (3351) {735} [5]
Ethnicity		Hanging/asphyxiation	568 (497) {455} [45]
White	800 (1648) {493} [1457]	Other method	1235 (2734) {373} [90]
Mixed	680 (550) {499} [37]	Drowning	850 (879) {454} [5]
Asian	651 (800) {517} [43]	Self-poisoning*	
Black	751 (771) {563} [19]	Major tranq. & mood stabilisers	469 (403) {487} [40]
Other	580 (270) {518} [22]	Benzodiazepines & other sedatives	655 (593) {503} [65]
Not known	1471 (4015) {427} [45]	Tricyclic antidepressants	1140 (1836) {664} [26]
Residence***		All other antidepressants	1006 (3087) {496} [78]
Home not alone	933 (1905) {547} [894]	Any pure paracetamol (compounds)	746 (725) {576} [253]
Home alone	823 (1600) {503} [297]	Other non-opiate analgesics	988 (2930) {489} [58]
Lodging/hostel	695 (1032) {495} [152]	Opiate drugs only	715 (1851) {486} [60]
Institution	320 (378) {185} [87]	All other drugs	699 (756) {543} [141]
Not fixed/ Not known	522 (1634) {187} [193]	Drug not known	1449 (2120) {504} [16]
Morbidity**		Multiple incl any paracetamol	756 (1297) {550} [208]
No	795 (1223) {550} [404]	Multiple incl tricyclics	1944 (3409) {538} [21]
Single physical condition	797 (950) {600} [127]	Multiple incl tricyclics & paracetamol	1038 (1131) {613} [16]
Single mental health condition	1004 (2059) {575} [447]	Multiple excl tricyclics & paracetamol	786 (1445) {512} [155]
Multiple physical conditions	645 (481) {481} [36]		
Physical and mental conditions	1074 (2350) {558} [207]		
Not known	487 (1504) {159} [402]		

* $p<0.05$ ** $p<0.01$ *** $p<0.001$; Note: the differences in means are tested with Kruskal-Wallis and Mann-Whitney statistical tests
